# Empagliflozin reduces liver fibrosis by restoring catechol-*O*-methyltransferase activity associated with magnesium levels

**DOI:** 10.1038/s41598-025-12813-x

**Published:** 2025-07-26

**Authors:** Yoshihiro Hayashi, Emi Kawakita, Naoki Kumashiro, Hiroshi Iijima, Daisuke Koya, Keizo Kanasaki

**Affiliations:** 1 Faculty of Medicine, Internal Medicine 1, Shimane University, 89-1 Enya-Cho, Izumo, Shimane 693-8501 Japan; 2 Department of Diabetes and Endocrinology, Kanazawa Medical University, 1-1 Daigaku, Uchinada-Machi, Kahoku District, Ishikawa, 920-0293 Japan; 3 School of Pharmacy, Nihon University, 7-7-1 Narashinodai, Funabashi, Chiba 274-8555 Japan; 4 CBI Research Institute, 3-11-1 Shibaura, Minato-Ku, Tokyo, 108-0023 Japan; 5 Division of Anticipatory Molecular Food Science and Technology, Kanazawa Medical University, Medical Research Institute, 1-1 Daigaku, Uchinada-Machi, Kahoku District, Ishikawa, 920-0293 Japan; 6 The Center for Integrated Kidney Research and Advance, Faculty of Medicine, Shimane University, 89-1 Enya-Cho, Izumo, Shimane 693-8501 Japan

**Keywords:** Diabetes mellitus, Magnesium, Catechol-*O*-methyltransferase, Sodium–glucose cotransporter 2 inhibitor, Liver fibrosis, Metabolic disorders, Endocrine system and metabolic diseases

## Abstract

Catechol-*O*-methyltransferase (COMT), a magnesium (Mg)-dependent enzyme, metabolises catecholamines. Diabetic patients exhibit hypomagnesemia and sympathetic overactivity compared with non-diabetics. Sodium–glucose cotransporter 2 (SGLT2) inhibitors increase serum Mg levels in diabetic patients. Sympathetic overactivity is associated with diabetic complications; however, the entire mechanism has not been elucidated. Type 2 diabetes model BKS^*db/db*^ male mice were fed either a control or an empagliflozin-supplemented diet. Mg^2+^ concentrations, catecholamines, and COMT activity and protein levels were measured. Human Kupffer cells (hKCs) were incubated with norepinephrine (NE) and normetanephrine (NMN), and interleukin (IL)-6 concentrations were quantified. In non-diabetic mice, Mg^2+^ deficiency was associated with decreased liver COMT activity. In diabetic mice, empagliflozin, an SGLT2 inhibitor, increased plasma Mg^2+^ levels and elevated the hepatic NMN/(NE + NMN) ratio. Liver COMT activity was suppressed in diabetic mice; however, empagliflozin restored COMT activity without altering COMT protein expression. Empagliflozin ameliorated fibrosis and IL-6 levels in the liver. In hKCs, NE stimulated IL-6 production, which was attenuated by NMN preincubation. We demonstrated that SGLT2 inhibitors regulate sympathetic activity by enhancing COMT activity in diabetic mice. These findings suggest a new potential health benefit of SGLT2 inhibitors.

## Introduction

Sodium–glucose cotransporter 2 (SGLT2) inhibitors have been shown to exert a glucose-independent cardiorenal protective effect in diabetic patients^1,2^. More recently, similar effects have been demonstrated in non-diabetic patients^3,4^. Hyperinsulinemia and insulin resistance are associated with sympathetic activation, and approximately half of diabetic patients exhibit sympathetic dysfunction^5,6^. Sympathetic activation is related not only to diabetes but also to other diseases, such as hypertension, heart failure, and chronic kidney disease^7–9^. Sympathetic overactivity is a risk factor for cardiovascular and kidney disease^10,11^. In contrast, its effect on other diabetic complications remains unclear. It is important to control the sympathetic nervous system to prevent diabetic complications; however, few treatments are available to ameliorate sympathetic activity. A previous study reported that the crosstalk between the heart, kidney, and central nervous system causes systemic sympathetic activation via the afferent renal nerve; SGLT2 inhibitors improve this process^12^. Although inhibition of sympathetic activity may be an underlying mechanism by which SGLT2 inhibitors prevent diabetic complications, the details remain poorly understood.

Catechol-*O*-methyltransferase (COMT) is an enzyme responsible for the metabolism of catecholamines, converting them into metabolites with reduced biological activity through methylation. The methyl group is donated by *S*-adenosylmethionine (SAM), which is subsequently converted into *S*-adenosylhomocysteine (SAH). COMT requires divalent magnesium ion (Mg^2+^) as the essential cofactor. Mg^2+^, the second most abundant intracellular cation, functions as a cofactor in more than 300 enzymatic reactions^13^. In the context of COMT, Mg^2+^ facilitates the interaction between the catechol oxygen atom and SAM, thereby promoting their efficient reaction^14^. Our previous report revealed that dietary Mg insufficiency induces salt-sensitive hypertension in mice that is associated with reduced kidney COMT activity^15^, indicating an association between COMT activity and plasma Mg^2+^ levels. COMT activity is affected not only by Mg^2+^ but also by other factors, including genetic factors. Single nucleotide polymorphisms (SNPs) are related to COMT activity, and one of the most common SNPs in the COMT gene is rs4680 (Val158Met). The rs4680 polymorphism has three genotypes: Val/Val, Val/Met, and Met/Met. Compared with the Met/Met genotype, the Val/Val genotype has approximately 1.4-fold greater COMT activity^16^. The lowest COMT activity genotype, Met/Met, is associated with an increased risk of acute coronary events and hypertension^17,18^. Furthermore, genome-wide association studies indicated that rs4680 may be a risk variant for type 2 diabetes mellitus (T2DM)^19^.

Diabetic patients exhibit a high prevalence of hypomagnesemia^20^. Hypomagnesemia is associated with the development of T2DM via mechanisms such as increased insulin resistance^6,21^. Chronic Mg deficiency is related to the development and exacerbation of many diseases, such as heart failure, ischaemic heart disease, hypertension, metabolic syndrome, osteoporosis, depression, migraines, and asthma^22^. In a meta-analysis, SGLT2 inhibitors were shown to increase serum Mg levels in diabetic patients^23^; however, the role of the SGLT2 inhibitor-induced elevation of serum Mg levels in COMT activity has not yet been investigated. In this study, we aimed to elucidate the effect of SGLT2 inhibitor empagliflozin on COMT activity through elevated serum Mg and the impact on organ protection via reduced sympathetic nerve activity.

## Results

### Blood pressure and pulse rate did not differ between the BKS^***db/db***^ control and empagliflozin-treated groups

**Fig. 1:**
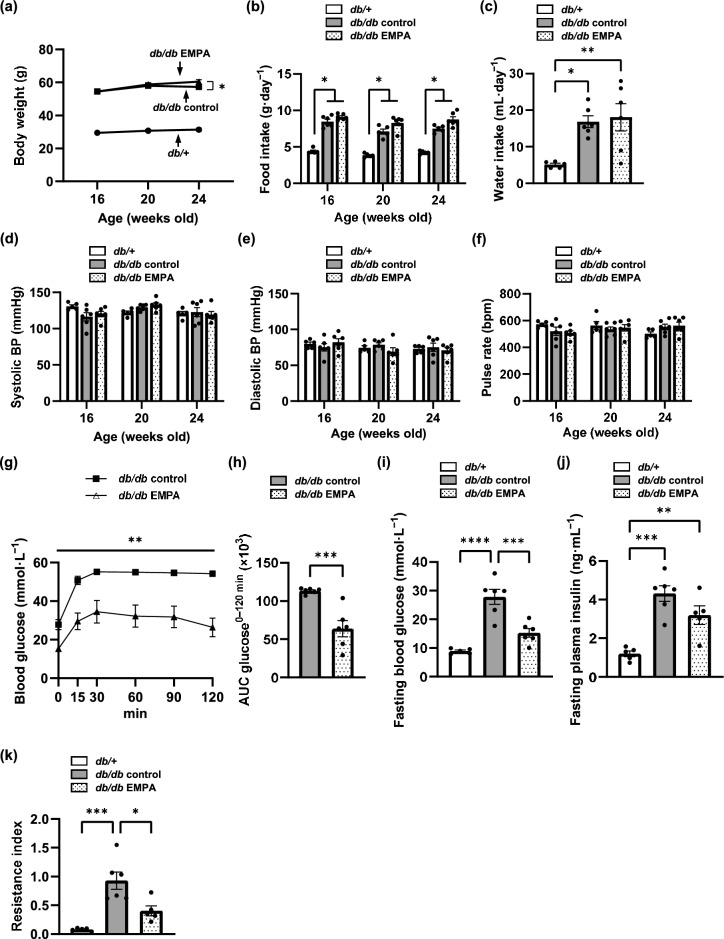
Empagliflozin did not affect blood pressure or pulse rate and ameliorated glucose tolerance. (**a**) Body weight was measured every four weeks from baseline (16 weeks old) in BKS^*db/*+^ (*db/* +), BKS^*db/db*^ (*db/db*) control, and *db/db* empagliflozin (EMPA) mice. (**b**) Food intake was measured every four weeks from baseline in *db/* +, *db/db* control, and *db/db* EMPA mice. (**c**) Water intake was measured in *db/* +, *db/db* control, and *db/db* EMPA mice at 24 weeks of age using a metabolic cage. (**d**–**f**) Systolic blood pressure (BP), diastolic BP, and pulse rate were measured noninvasively every four weeks from baseline using a tail-cuff system and were evaluated by an average of eight measurements in *db/* +, *db/db* control, and *db/db* EMPA mice. (**g**) An intraperitoneal glucose tolerance test was performed in *db/db* control and *db/db* EMPA mice at 24 weeks of age after 16 h of fasting. Glucose (1 g·kg^−1^ body weight of D-(+)-glucose) was injected intraperitoneally. Blood glucose was measured at 0, 15, 30, 60, 90, and 120 min after glucose injection. (**h**) The area under the curve (AUC) of blood glucose from 0 to 120 min was compared between *db/db* control and *db/db* EMPA mice. (**i**, **j**) Fasting blood glucose and fasting plasma insulin levels were measured in *db/* +, *db/db* control, and *db/db* EMPA mice after 16 h of fasting at 24 weeks of age. (**k**) The resistance index was calculated as follows: resistance index = glucose (mmol·L^−1^) × insulin (mU·L^−1^)/22.5. *db/* +, n = 5. *db/db* control, n = 6. *db/db* EMPA, n = 6. The data are presented as the mean ± SEM. The data were analysed using one-way ANOVA followed by Tukey’s test (**a**–**f**, **i**–**k**) and the Student’s *t* test (**g**, **h**). *p < 0.05, **p < 0.01, ***p < 0.001, ****p < 0.0001.

The body weights of BKS^*db/db*^ (*db/db*) mice were greater than those of BKS^*db/*+^ (*db/* +) mice, and at 24 weeks of age, those of BKS^*db/db*^ SGLT2 inhibitor empagliflozin-treated *(db/db* EMPA) mice were significantly greater than those of BKS^*db/db*^ control (*db/db* control) mice (Fig. 1a). The *db/db* mice consumed more food and water than the *db/* + mice; in contrast, food and water intake did not differ between the two groups of *db/db* mice (Fig. 1b and c). Systolic blood pressure (BP), diastolic BP, and pulse rate also did not differ among the three groups (Fig. 1d–f). The intraperitoneal glucose tolerance test (IPGTT) revealed that empagliflozin significantly decreased blood glucose at fasting and after glucose loading in diabetic mice (Fig. 1g–i). Fasting plasma insulin levels in *db/db* control mice were significantly higher than those in *db/* + mice; empagliflozin tended to decrease these levels (Fig. 1j). The resistance index, indicating insulin resistance, was significantly decreased by empagliflozin (Fig. 1k). Heart and kidney weights were greater in the two groups of *db/db* mice; however, as reported previously^24^, liver weight was increased in *db/db* control mice than in *db/* + mice, which was decreased with empagliflozin treatment (Supplementary Fig. S1).

### Hypomagnesemia decreased liver COMT activity in DBA/2J mice: empagliflozin elevated plasma Mg^2+^ concentration in *db/db* mice

**Fig. 2:**
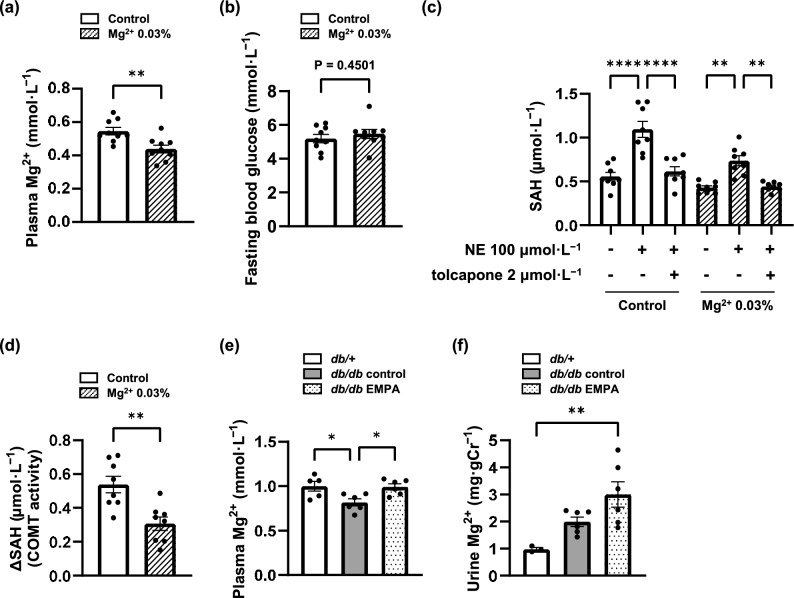
Dietary magnesium (Mg^2+^) deficiency decreased plasma Mg^2+^ concentration and liver catechol-*O*-methyltransferase (COMT) activity in non-diabetic mice, and empagliflozin increased plasma Mg^2+^ concentration in diabetic mice. (**a**) Male DBA/2J mice were fed a control diet (1,000 mg Mg^2+^·kg^−1^) or a 0.03% Mg^2+^ diet (300 mg Mg^2+^·kg^−1^) for 4 weeks, and plasma Mg^2+^ concentrations were measured. (**b**) Fasting blood glucose was measured after 16 h of fasting at 11 weeks of age. (**c**) A methyltransferase assay kit, which detects the *S*-adenosylhomocysteine (SAH) reaction product, was used to measure the SAH concentration in the liver. Norepinephrine (NE) was used as a substrate, and tolcapone was used as a COMT inhibitor. (**d**) The difference in liver SAH between samples with and without NE (ΔSAH), which represents liver COMT activity, was calculated. (**e**) Plasma Mg^2+^ concentrations were measured in *db/* +, *db/db* control, and *db/db* EMPA mice at 24 weeks of age. (**f**) Urinary Mg^2+^ excretion was measured in *db/* +, *db/db* control, and *db/db* EMPA mice at 24 weeks of age. DBA/2J control, n = 9. DBA/2J 0.03% Mg^2+^, n = 9. *db/* +, n = 5. *db/db* control, n = 6. *db/db* EMPA, n = 6. The data are presented as the mean ± SEM. The data were analysed using the Student’s *t* test (**a**, **b**, **d**) and one-way ANOVA followed by Tukey’s test (**c**, **e**, **f**). *p < 0.05, **p < 0.01, ****p < 0.0001.

First, we measured plasma Mg^2+^ concentration and liver COMT activity to confirm their association using DBA/2J (DBA) mice. In DBA mice, dietary Mg^2+^ insufficiency induced a decrease in plasma Mg^2+^ concentration (Fig. 2a). Hypomagnesemia did not affect fasting blood glucose (Fig. 2b). We evaluated COMT activity using a methyltransferase assay kit, which considers the difference in SAH concentration with or without substrate as COMT activity. Norepinephrine (NE) was used as a substrate for COMT. Additionally, a group with tolcapone was assessed to confirm whether the elevation of SAH was caused by COMT alone. The SAH concentration was elevated with NE incubation in the livers of DBA mice; tolcapone decreased the SAH concentration to the same level as that in the group without NE (Fig. 2c). Therefore, the increase in SAH concentration with NE was caused by COMT. The difference in SAH concentration with and without NE (ΔSAH), which represents COMT activity, was significantly decreased in the dietary Mg^2+^-insufficient mice (Fig. 2d). This finding demonstrated that the low plasma Mg^2+^ level caused low COMT activity in the liver. Next, to confirm the effect of empagliflozin on Mg^2+^, we measured plasma Mg^2+^ concentration and urinary Mg^2+^ excretion in BKS mice. Plasma Mg^2+^ concentrations decreased in *db/db* control mice compared with those in *db/* + mice. As in previous clinical trials^23^, empagliflozin increased plasma Mg^2+^ concentrations in diabetic mice (Fig. 2e). Although reducing urinary Mg^2+^ excretion has been considered one of the mechanisms of serum Mg^2+^ elevation by SGLT2 inhibitors^25^, urinary Mg^2+^ excretion did not differ between *db/db* control and *db/db* EMPA mice in this study (Fig. 2f).

### Liver catecholamine metabolism was promoted by empagliflozin

**Fig. 3:**
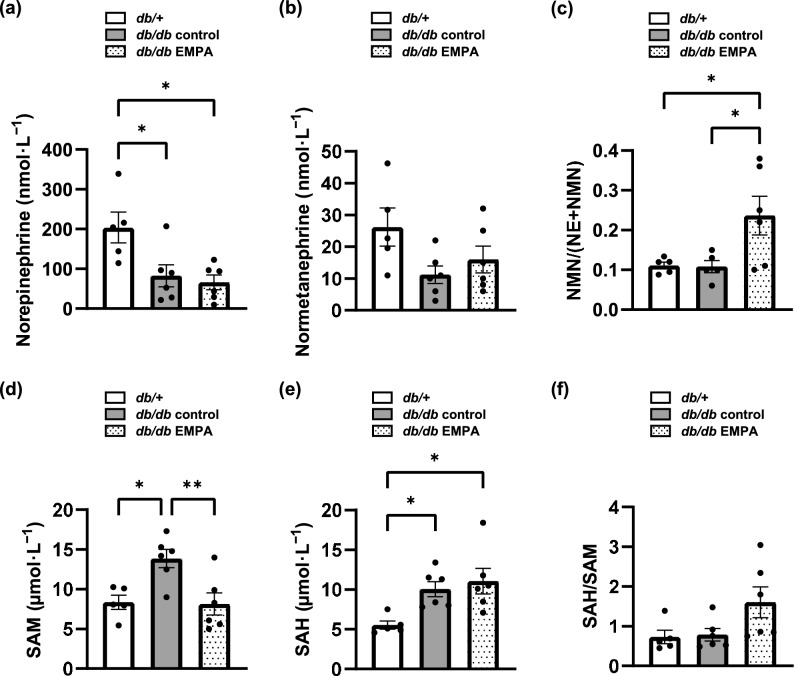
Empagliflozin (EMPA) promoted norepinephrine (NE) metabolism in the livers of diabetic mice. (**a**) Liver NE and (**b**) normetanephrine (NMN) concentrations were measured in *db/* +, *db/db* control, and *db/db* EMPA mice. (**c**) The ratios of NMN to total concentrations of NE and NMN were calculated. (**d**) Liver *S*-adenosylmethionine (SAM) and (**e**) *S*-adenosylhomocysteine (SAH) concentrations were measured. (**f**) The ratios of SAH to SAM were calculated. *db/* +, n = 5. *db/db* control, n = 6. *db/db* EMPA, n = 6. The data are presented as the mean ± SEM. The data were analysed using one-way ANOVA followed by Tukey’s test. *p < 0.05, **p < 0.01.

The liver plays a major role in the inactivation of circulating catecholamines^26^. In the livers of diabetic mice, empagliflozin administration tended to decrease NE concentrations and to increase normetanephrine (NMN) concentrations (Fig. 3a and b). Unexpectedly, NE levels were higher in *db/* + mice than in *db/db* control mice. The ratio of NMN to the total concentration of NE and NMN significantly increased with empagliflozin (Fig. 3c). These findings suggest that empagliflozin promoted the metabolism of NE to NMN in diabetic mice. SAM was converted to SAH when COMT metabolised catecholamines to methylated metabolites. In *db/db* control mice, the liver SAM concentration was significantly higher than in *db/* + mice. Empagliflozin significantly reduced the liver SAM concentration in *db/db* mice and tended to increase the SAH/SAM ratio (Fig. 3d–f), suggesting that COMT-mediated NE metabolism was promoted by empagliflozin. The levels of catecholamines and their metabolites in the plasma did not differ between the two groups of diabetic mice (Supplementary Fig. S2). NE and NMN concentrations in the heart and kidney showed trends similar to those in the liver, although the differences were not statistically significant (Supplementary Fig. S3 and S4).

### Empagliflozin increased liver COMT activity and ameliorated liver fibrosis

**Fig. 4:**
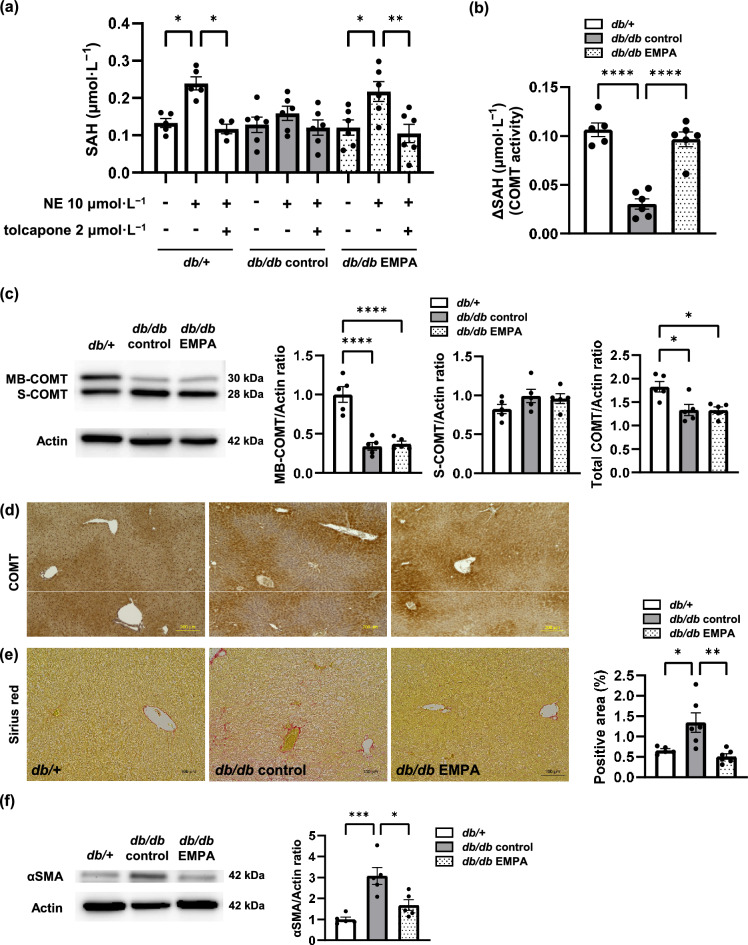
Empagliflozin (EMPA) increased liver catechol-*O*-methyltransferase (COMT) activity and ameliorated liver fibrosis in diabetic mice. (**a**) A methyltransferase assay kit, which detects the *S*-adenosylhomocysteine (SAH) reaction product, was used to measure the liver SAH concentration in *db/* +, *db/db* control and *db/db* EMPA mice. Norepinephrine (NE) was used as a substrate, and tolcapone was used as a COMT inhibitor. (**b**) The difference in liver SAH between samples with and without NE (ΔSAH), which represents liver COMT activity, was calculated. (**c**) Liver membrane-bound (MB), soluble (S), and total COMT protein levels were analysed using western blotting. The data were normalised to those of β-actin. Original blots are presented in Supplementary Fig. S5. (**d**) Immunohistochemistry analysis was performed on the liver. The scale bar represents 200 µm. (**e**) Sirius red staining was performed on the liver. The scale bar represents 100 µm. (**f**) Liver α-smooth muscle actin (αSMA) protein levels were analysed using western blotting. Original blots are presented in Supplementary Fig. S6. *db/* +, n = 5. *db/db* control, n = 6. *db/db* EMPA, n = 6. The data are presented as the mean ± SEM. The data were analysed using one-way ANOVA followed by Tukey’s test. *p < 0.05, **p < 0.01, ***p < 0.001, ****p < 0.0001.

To confirm whether the increase in the NMN ratio to the total concentration of NE and NMN was caused by COMT, we evaluated COMT activity in the liver. As in the DBA mouse experiment, we confirmed the increase in the SAH concentration with NE incubation and the decrease in SAH with tolcapone (Fig. 4a). COMT activity, represented by ΔSAH, was decreased in *db/db* control mice and ameliorated by empagliflozin (Fig. 4b). To confirm that the difference in the elevation of SAH between the two diabetic mouse groups did not depend on the COMT protein level, we performed western blotting. COMT has two isoforms: membrane-bound COMT (MB-COMT) and soluble COMT (S-COMT)^15^. Western blotting revealed a significant decrease in MB-COMT in diabetic mice compared with non-diabetic mice; however, no differences were observed in MB-COMT, S-COMT, or total COMT protein levels between *db/db* control and *db/db* EMPA mice (Fig. 4c). Immunohistochemistry analysis revealed a mottled pattern of COMT protein expression in diabetic mice (Fig. 4d). Sirius red staining showed that liver fibrosis was significantly exacerbated in *db/db* control mice compared with *db/* + mice, and this was ameliorated by empagliflozin (Fig. 4e). The protein expression of α-smooth muscle actin (αSMA) was significantly elevated in the livers of *db/db* control mice compared with *db/* + mice; empagliflozin significantly attenuated αSMA expression (Fig. 4f).

### NMN reduced NE-induced Interleukin-6 production in human Kupffer cells

**Fig. 5:**
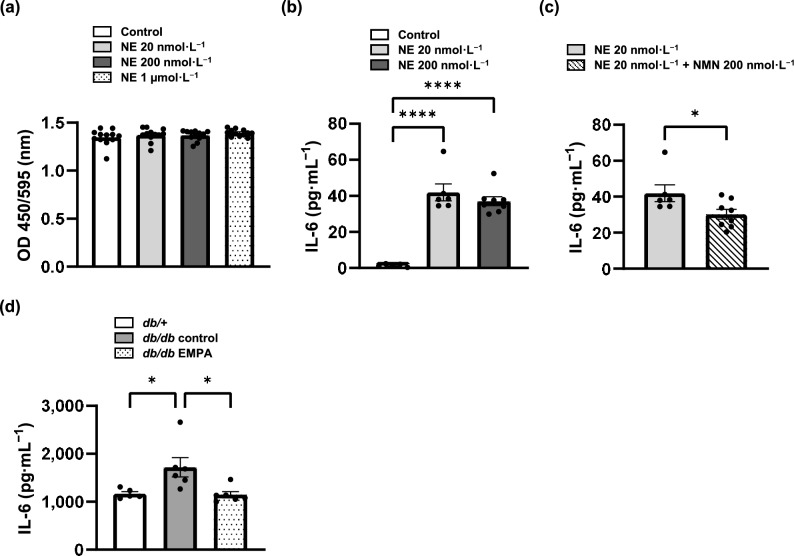
Norepinephrine (NE) stimulated IL-6 production in human Kupffer cells (hKCs), and normetanephrine (NMN) inhibited NE-induced IL-6 production. (**a**) The BrdU cell proliferation assay in LX-2 cells (n = 12 per group). (**b**) IL-6 production in hKCs exposed to NE (20 nmol·L^−1^ or 200 nmol·L^−1^) for 16 h was measured compared with that in the control group (n = 8 per group). (**c**) IL-6 production in hKCs exposed to NE (20 nmol·L^−1^) for 16 h was compared with that in the group subjected to a 2-h NMN (200 nmol·L^−1^) preincubation prior to the 16-h NE (20 nmol·L^−1^) exposure (n = 8 per group). (d) Liver IL-6 concentration in 1 mg of liver homogenate. *db/* +, n = 5. *db/db* control, n = 6. *db/db* EMPA, n = 6. The data are presented as the mean ± SEM of independent experiments. The data were analysed using one-way ANOVA followed by Tukey’s test (**a**, **b**, **d**) and the Student’s *t* test (**c**). *p < 0.05, ****p < 0.0001.

First, we performed an in vitro experiment using the hepatic stellate cell (HSC) line LX-2 cells to evaluate the association between COMT activity and liver fibrosis. Unexpectedly, in the BrdU assay, NE did not promote proliferation in LX-2 cells (Fig. 5a). Next, we turned our attention to Kupffer cells (KCs), which are known to contribute to liver fibrosis through paracrine mechanisms involving interleukin (IL)−6 production^27^. A previous study reported that NMN displayed 1/600 activity of NE on blood pressure^28^, suggesting that NMN may be an endogenous competitive inhibitor of NE. Based on this finding, we investigated whether NMN could attenuate the effects of NE. In human Kupffer cells (hKCs), NE promoted IL-6 production (Fig. 5b), whereas NMN significantly reduced NE-induced IL-6 production (Fig. 5c). In the liver, the IL-6 concentrations were significantly higher in *db/db* control mice than in *db/* + mice, and empagliflozin significantly reduced these levels (Fig. 5d).

## Discussion

In this study, we aimed to reveal the interaction between SGLT2 inhibitor-induced amelioration of hypomagnesemia and COMT activity, and those impacts on sympathetic activity. COMT metabolises catecholamines, and its activity depends on Mg^2+^. Diabetic patients exhibit more hypomagnesemia than non-diabetic patients^20^, and the sympathetic nervous system is activated in diabetic patients. Sympathetic activation induces organ damage and dysfunction, such as heart or kidney disease^10,11^. SGLT2 inhibitors cure hypomagnesemia in diabetic patients^23^. Based on previous reports, we hypothesised that SGLT2 inhibitors ameliorate sympathetic overactivation by increasing COMT activity by increasing plasma Mg^2+^ levels. Therefore, this study focused on the association between the regulation of sympathetic activity by COMT and diabetic complications.

In the present study, we employed BKS^*db/db*^ mice, which are among the most widely used models for investigating T2DM and its complications. We have previously reported comparative analyses of organ fibrosis using BKS^*db/db*^ and CD-1^*db/db*^ mice^29^. Although BKS^*db/db*^ mice exhibit β-cell dysfunction, our prior data indicate that they do not develop ketoacidosis at 24 weeks of age and maintain insulin secretion at this stage. Based on our accumulated data and comprehensive understanding of the baseline characteristics and phenotypes of BKS^*db/db*^ mice, we selected this strain for the current study. At the same time, it remains difficult to determine whether our findings are applicable to other strains of *db/db* mice or to other species, including humans.

The following observations were obtained: (1) Hypomagnesemia decreases liver COMT activity in non-diabetic mice; (2) empagliflozin elevates plasma Mg^2+^ levels in diabetic mice; (3) empagliflozin-induced increases in plasma Mg^2+^ levels result in increased liver COMT activity; (4) in the liver, NE metabolism to NMN is promoted in association with increased liver COMT activity in diabetic mice; (5) NE stimulates IL-6 production in hKCs, which is competitively inhibited by NMN; and (6) empagliflozin reduced IL-6 levels in the liver in diabetic mice. These results suggest that SGLT2 inhibitors regulate sympathetic activity by promoting NE metabolism through increased COMT activity, and that the inhibition of IL-6 production via sympathetic regulation contributes to their liver-protective effects. Indeed, hypomagnesemia has been shown to be relevant to the pathogenesis of liver fibrosis^30^.

COMT regulates sympathetic activity by metabolising catecholamines. Mg^2+^ plays an important role in catecholamine metabolism by COMT by facilitating the binding of catecholamines within the catalytic pocket and activating the catechol oxygen atom to initiate a nucleophilic attack on the methyl group of SAM^14^. In BKS mice, the plasma Mg^2+^ level was lower with diabetes; this Mg^2+^ level was increased by empagliflozin, similar to a previous study in humans^23^. The mechanisms by which SGLT2 inhibitors increase serum Mg^2+^ levels include increased reabsorption of Mg^2+^ in the kidney, increased intestinal absorption of Mg^2+^, and an increased shift of Mg^2+^ from the intracellular to the extracellular compartment^25^. In this study, empagliflozin did not affect urinary Mg^2+^ excretion. Hence, the mechanisms responsible for the increase in plasma Mg^2+^ levels could be increased intestinal absorption and/or an extracellular shift. Furthermore, our study showed that empagliflozin increased COMT activity in the liver, where it increased the proportion of NMN and decreased the SAM concentration. These results suggest that empagliflozin enhances COMT activity, which promotes catecholamine metabolism. As the COMT protein levels in the liver did not differ between the *db/db* control and EMPA mice, we confirmed that the COMT protein level did not affect the increase in COMT activity in the liver. Moreover, the DBA mouse experiment revealed that hypomagnesemia induced a decrease in liver COMT activity in non-diabetic mice, which showed that plasma Mg^2+^ levels impacted liver COMT activity regardless of blood glucose. These results indicated that the elevation of plasma Mg^2+^ levels by empagliflozin led to an increase in COMT activity in the liver and the regulation of sympathetic activity. Unexpectedly, NE concentrations in the liver were higher in *db/* + mice than in *db/db* control mice. The activities of catecholamine-metabolising enzymes other than COMT could have contributed to the difference in NE levels. Additionally, a previous study reported that NE sensitivity in the caudal artery was significantly greater in diabetic rats than in non-diabetic rats^31^. Based on this finding, NE sensitivity in the liver in *db/db* control mice could have been greater than in *db/* + mice in the present study.

Metabolic dysfunction-associated steatotic liver disease (MASLD), previously called non-alcoholic fatty liver disease (NAFLD), is a diabetic complication; approximately 65% of people with T2DM have NAFLD^32^. NAFLD causes hepatic inflammation and fibrosis, and it increases the risk of liver-related complications such as cirrhosis, liver failure, hepatocellular carcinoma, and death^33^. According to a meta-analysis, NAFLD is related to increased overall mortality^34^. Therefore, revealing the mechanisms of MASLD and discovering effective treatments are important clinical issues. Inflammatory cytokines are one of the reasons for the development and exacerbation of NAFLD^35^. A meta-analysis revealed that inflammatory cytokines such as IL-1β, IL-6, and tumour necrosis factor (TNF)-α are associated with the risk of NAFLD^36^. Inflammatory cytokines are produced by many kinds of cells, including immune cells. The representative macrophages in the liver are mainly Kupffer cells, which are activated by various factors, such as lipopolysaccharides and metabolic dysfunction, including lipid and iron metabolism^37^. Activated KCs play a role in immune responses by releasing inflammatory cytokines; conversely, persistent activation of KCs causes MASLD or MASH^37^. In terms of inflammatory cytokines in the liver, patients with non-alcoholic steatohepatitis (NASH), currently called metabolic dysfunction-associated steatohepatitis (MASH), present higher expression of hepatic IL-6 than normal individuals; moreover, a positive correlation has been observed between hepatocyte IL-6 expression and the degree of inflammation and stage of fibrosis^38^.

HSCs, the key drivers of liver fibrosis, are activated by oxidative stress and inflammatory signals. Activated HSCs induce enhanced proliferation, differentiation into myofibroblast-like cells, chemotaxis, fibrogenesis, and the production of extracellular matrix remodelling enzymes^39^. In our study, NE did not directly promote the proliferation of LX-2 cells. Therefore, we focused on paracrine signalling. A previous study revealed that HSCs treated with IL-6 demonstrated an upregulation of αSMA and Col1a mRNA levels and increased αSMA protein levels, which were reduced by inhibition of MAPK or JAK/STAT3 signalling pathways^40^. Furthermore, the coculture of primary KCs and HSCs induced a more activated phenotype, which was inhibited by anti-IL-6 and siRNA-IL-6^27^, indicating that KCs activate HSCs via the production of IL-6 and lead to liver fibrosis.

According to a previous report on the relationship between NE and KCs, NE activates KCs and induces the release of inflammatory cytokines from KCs^41,42^. In our study, empagliflozin increased the ratio of NMN to the total concentration of NMN and NE in the liver, indicating that the ratio of NE decreased. NMN is an inactive metabolite of NE. A previous study reported that NMN is 1/600 as active as NE^28^, suggesting that NMN binds adrenergic receptors and could competitively inhibit NE binding to adrenergic receptors. Therefore, we focused on the differences in the activation of KCs by NE and NMN. NE promoted the production of IL-6 in KCs in vitro, whereas NMN inhibited it. These findings suggest that NMN could prevent liver fibrosis by inhibiting the activation of HSCs through the reduction of IL-6 production in KCs.

A previous study revealed that SGLT2 inhibitors lowered blood pressure^43^. The mechanisms by which SGLT2 inhibitors lower blood pressure include weight loss, the reduction of circulating plasma volume through osmotic and natriuretic diuresis, and the regulation of the sympathetic nervous system^44^. Conversely, in our study, empagliflozin neither affected blood pressure nor reduced catecholamine levels in the blood. Fundamentally, BKS^*db/db*^ mice did not present prominent hypertension.

A limitation of the present study is that the mechanism by which plasma Mg^2+^ increases liver COMT activity was not confirmed. We revealed that the elevation of plasma Mg^2+^ levels by SGLT2 inhibitors caused increased liver COMT activity; however, we did not elucidate how Mg^2+^ impacted COMT and accelerated its enzymatic reaction at the molecular level. Another limitation is the influence of other organs on catecholamines in addition to the liver. Many organs utilise and metabolise catecholamines, and organs other than the liver could have affected the measured catecholamine levels. Especially, the brain is one of the most important organs related to COMT activity. Differences in COMT activity are associated with cognitive phenotypes, psychiatric disorders, and changes in brain activation and structure^45^; however, brain COMT activity was not measured in this study. In addition, since the activities of catecholamine-metabolising enzymes other than COMT were not assessed, their potential impact on the concentrations of NE, NMN, SAM, and SAH remains unclear. The potential difference in NE sensitivity between *db/* + and *db/db* mice was not investigated and further study should be required. Furthermore, the effect of blood glucose is also a limitation. Empagliflozin lowered blood glucose levels in comparison to the *db/db* control mice. Since SGLT2 inhibitors were the only agents that lowered blood glucose and elevated plasma Mg^2+^ levels, we could not establish a group without reduced glucose levels. Therefore, we could not have evaluated the direct effect of blood glucose on COMT activity with SGLT2 inhibitors. Hence, we investigated the association between plasma Mg^2+^ and liver COMT activity in non-diabetic mice to exclude the effect of blood glucose on liver COMT activity. The DBA mouse experiments revealed that hypomagnesemia caused a decrease in liver COMT activity, which indicated that plasma Mg^2+^ levels affected liver COMT activity independently of blood glucose levels. This result suggests that SGLT2 inhibitors may increase liver COMT activity by elevating plasma Mg^2+^ levels, even eliminating their glycaemic effect. Finally, our analysis included an insufficient number of animals due to budget constraints, which prevented us from achieving statistically significant results for NE, NMN, SAH, and the SAH/SAM ratio in the liver, which showed similar trends in the liver, kidney, and heart. Significance may have been observed in the kidney and heart if more samples had been obtained.

In conclusion, our study revealed that SGLT2 inhibitors regulate sympathetic activity by increasing COMT activity associated with the correction of hypomagnesemia and may be involved in preventing liver fibrosis by inhibiting IL-6 production in KCs.

## Methods

### Ethical statement

We confirmed that all methods were performed in accordance with relevant guidelines and regulations. All animal experiments were reported as described by the ARRIVE guidelines. The animal experiments were performed following protocols (IZ2-49, IZ3-76, IZ5-11, IZ6-30) that were approved by the institutional animal care and use committee (IACUC) of Shimane University.

### Housing and euthanasia of mice

The mice were housed in a temperature-controlled room with an automatic 12-h light/dark cycle. Euthanasia was performed in a 2-step process using isoflurane (Viatris, Canonsburg, PA). Mice were first rendered unconscious through 3% isoflurane and then subsequently subjected to cardiac puncture for humane termination.

### Animal experiments

We obtained ten-week-old male BKS^*db/*+^ (BKS.Cg-*Dock7*^*m*^ + */* + *Lepr*^*db*^/J) mice and T2DM model BKS^*db/db*^ mice from The Jackson Laboratory Japan (Tokyo, Japan). The mice were fed a control diet (obtained from Boehringer Ingelheim, Germany) (BKS^*db/*+^, n = 6; BKS^*db/db*^, n = 6) or an SGLT2 inhibitor empagliflozin-supplemented diet (1.5 mg empagliflozin in a 10 g diet, obtained from Boehringer Ingelheim, Germany) (BKS^*db/db*^, n = 6) starting at 16 weeks of age. All the mice were provided free access to food and water. One of the BKS^*db/*+^ mice died because of failure of the water-serving system. Body weight, food intake, water intake, blood pressure, pulse rate, and blood glucose were measured every four weeks beginning at 16 weeks of age. IPGTT and 24-h urine collection were performed at 24 weeks of age. BP and pulse rate were measured noninvasively using a tail-cuff system (Softron, Tokyo, Japan) and were evaluated by an average of eight measurements. Blood glucose was measured using GLUTESTMINT II (SANWA KAGAKU KENKYUSHO, Aichi, Japan). The mice were sacrificed at 24 weeks of age. Blood, heart, liver, and kidney samples were collected for analysis.

For the Mg deficiency experiments, we followed a previously reported protocol^15^. Male DBA/2J mice were purchased from CLEA Japan (Tokyo, Japan). The mice were fed a 0.1% Mg^2+^ diet (control diet, 1,000 mg Mg^2+^·kg^−1^, CLEA Japan) (n = 9) or a 0.03% Mg^2+^ diet (low-Mg diet, 300 mg Mg^2+^·kg^−1^, CLEA Japan) (n = 9) starting at 7 weeks of age. All the mice were provided free access to food and water. Fasting blood glucose was measured after 16 h of fasting at 11 weeks of age. The mice were sacrificed at 11 weeks of age. Blood and organ samples were collected to evaluate plasma Mg^2+^ concentration and COMT activity.

### IPGTT

The mice were fasted for 16 h, and an intraperitoneal injection of glucose (1 g·kg^−1^ body weight) (D-(+)-glucose, NACALAI TESQUE, Cat# 16806-25, Kyoto, Japan) was given to each mouse. Blood was collected from the tail vein. The blood glucose of each mouse was measured at 0, 15, 30, 60, 90, and 120 min postinjection of glucose. The area under the curve (AUC) of blood glucose was compared between the control group and the experimental group. The insulin concentration was measured using an ultrasensitive mouse insulin enzyme-linked immunosorbent assay (ELISA) kit (Morinaga BioScience, Cat# M1104, Kanagawa, Japan). The insulin resistance index (IRI) was calculated using a previously reported method (IRI = glucose (mmol·L^−1^) × insulin (mU·L^−1^)/22.5)^46^.

### Blood, urine, and organ lysate preparation

Blood was collected by cardiac puncture and placed into a heparinized tube. The blood was centrifuged at 1,500 rcf for 15 min and 4 °C, and the plasma was obtained. Twenty-four-hour urine was collected using a metabolic cage and centrifuged at 15,400 rcf for 15 min at 4 °C. The heart, liver, and kidney were homogenised with PowerMasher II (Nippi, Tokyo, Japan) using a RIPA Lysis Buffer System (Santa Cruz Biotechnology, Cat# sc-24948A, Dallas, TX) and centrifuged at 15,400 rcf for 15 min at 4 °C, after which the supernatant was collected.

### Protein concentration

A bicinchoninic acid (BCA) assay was used to measure the protein concentration of the organ lysates. A Pierce™ BCA Protein Assay (Thermo Fisher Scientific, Cat# 23225, Waltham, MA) was used to determine the protein concentration, which was adjusted for western blotting, COMT activity assays, and IL-6 ELISA.

### Plasma and urine Mg^2+^ concentration

The plasma and urine Mg^2+^ concentrations were measured using the Metallo Assay Magnesium Assay LS (Metallogenics, Cat# MG01M, Chiba, Japan). The urine was diluted 10 times for measurement.

### Preparation for catecholamine measurements

An equal volume of dilution solution (10 mmol·L^−1^ citric acid, 0.1 mg·mL^−1^ EDTA, 10 mmol·L^−1^ glutathione-reduced form, and 0.1% (v/v) Triton X-100) was added to 150 µL of plasma or organ lysate. The mixture was mixed vigorously with a vortex mixer and centrifuged at 10,000 rcf for 10 min at 4 °C. The supernatant was passed through a 0.45 µm pore size filter (Ultrafree®-MC Centrifugal Filter, Millipore, Cat# UFC30HV00, Darmstadt, Germany) by centrifugation at 1,000 rcf for 3 min.

### Catecholamine concentration

Tsunoda and Imai’s method was used to determine the concentration of catecholamines^47–49^. The test sample (80 µL) was first loaded on an Ion-exchange mini column (MF SCX 10 mm × 4 mm, Osaka Soda, Cat# 12415, Osaka, Japan) with A-solution (10 mmol·L^−1^ potassium phosphate, pH 7.4) at 1 mL·min^−1^, and the column was subsequently washed with A-solution for 3 min. The solvent was then switched to M-solution (72 mmol·L^−1^ potassium acetate, pH 3.2, 2.5 mmol·L^−1^ phosphatic acid, 4 mmol·L^−1^ sodium hexane sulfonate, and 5% (v/v) acetonitrile) with a flow rate of 0.5 mL·min^−1^. The captured amines were eluted and introduced onto an ODS column (Unison UK-C18, 15 cm × 4.6 mm, Imtakt, Kyoto, Japan) equilibrated with M-solution. The catecholamines were oxidised by a carbon electrode (650 mV). Then, the F-solution (105 mmol·L^−1^ ethylenediamine, 175 mmol·L^−1^ imidazole, 85.25% (v/v) acetonitrile, 4.75% (v/v) ethanol, and 10% (v/v) water) was mixed with a flow rate of 0.32 mL·min^−1^. The mixture was incubated at 95 °C in an online reaction coil. Finally, C-solution (0.25 mmol·L^−1^ *bis*[2,4,5-trichloro-6-(pentyloxycarbonyl)phenyl]oxalate, 150 mmol·L^−1^ H_2_O_2_, 110 mmol·L^−1^ TFA, 50% (v/v) dioxane, and 50% (v/v) ethyl acetate) was mixed (1.45 mL·min^−1^) with the oxidised reaction mixture, and the chemical luminescence was measured. The retention times (RTs) of NE, epinephrine (E), NMN, and metanephrine (MN) were 11.4 min, 13.0 min, 15.6 min, and 19.5 min, respectively.

### Quantification of SAM and SAH

The method developed by Tsuge was employed to quantify SAM and SAH in plasma, heart, liver, and kidney^50^. The sample (80 µL) was injected into an ODS reversed-phase chromatography column (TSKgel ODS-80T_M_, 25 cm × 4.6 mm, Tosoh Bioscience, Tokyo, Japan) equilibrated with a solution of 40 mmol·L^−1^ ammonium phosphate and 8 mmol·L^−1^ 1-heptanesulfonic acid sodium salt in 18% (v/v) methanol (pH adjusted to 3.0 with HCl). Isocratic elution was carried out at a flow rate of 1 mL·min^−1^ at 35 °C, and the absorbance at 260 nm was recorded. RT of SAH and SAM was 15.5 min and 17.8 min, respectively.

### COMT activity

COMT activity was evaluated using the MTase-Glo™ Methyltransferase Assay (Promega, Cat# V7601, Madison, WI), which monitors the formation of the reaction product SAH as a measure of methyltransferase activity. We used L-noradrenaline bitartrate monohydrate (Tokyo Chemical Industry, Cat# A0906, Tokyo, Japan) as the substrate and tolcapone (Sigma‒Aldrich, Cat# SML0150, St. Louis, MO) as a COMT inhibitor. The SAH standard was made following the manufacturer’s protocol. SAM was added to 4X reaction buffer (80 mmol·L^−1^ Tris buffer, 200 mmol·L^−1^ NaCl, 4 mmol·L^−1^ EDTA, 12 mmol·L^−1^ MgCl_2_, 0.4 mg·mL^−1^ BSA, and distilled water) to a final concentration of 20 µmol·L^−1^. Additionally, solutions with substrate or tolcapone were made. The solutions were diluted with distilled water to a final concentration of 1X reaction buffer, 20 µmol·L^−1^ SAM, 10 µmol·L^−1^ or 100 µmol·L^−1^ NE, and 2 µmol·L^−1^ tolcapone. A total of 10 µL of each solution was added to each well, except for the SAH standard, in a 96-well white plate with solid bottom. We mixed 1 µg of protein in 1X reaction buffer for each sample, and 10 µL of the mixture was added to each well except for the SAH standard. The plate was incubated for 1 h at 37 °C. Subsequently, 5 µL of 5X MTase-Glo™ Reagent was added to all the wells, and the plate was incubated for 30 min at room temperature. Finally, 25 µL of MTase-Glo™ detection solution was added to all the wells, and the plate was incubated for 30 min at room temperature. The luminescence was measured using a plate-reading luminometer. The difference in SAH concentration with and without substrate was evaluated as the COMT activity.

### Western blotting

An anti-COMT antibody (1:1000) (Proteintech, Cat# 14754-1-AP, Rosemont, IL) and an anti-α-smooth muscle actin antibody (1:1000) (abcam, Cat# ab5694, Cambridge, UK) were utilised for western blotting, with anti-β-actin antibody (1:2500) (MBL International, Cat# PM053-7, Tokyo, Japan) serving as a loading control. The protein lysate of the liver was mixed with SDS sample buffer and denatured by boiling at 95 °C for 5 min. Denatured protein lysates (15 µg) were separated on 4–20% Mini-PROTEAN® TGX™ Precast protein gels (Bio-Rad Laboratories, Cat# 4561095, Hercules, CA) by electrophoresis, and the samples were then transferred to a polyvinylidene fluoride (PVDF) membrane via the semidry method. The membrane was blocked for 30 min at room temperature with 5% (w/v) non-fat dry milk dissolved in Tris-buffered saline containing 0.1% (v/v) Tween 20 (TBST) and then incubated with a primary antibody overnight. After incubation, the membrane was washed three times with TBST and incubated with HRP-linked anti-rabbit secondary antibody (1:2500) (Cell Signaling Technology, Cat# 7074, Danvers, MA) for 1 h. Pierce™ ECL Western Blotting Substrate (Thermo Fisher Scientific, Cat# 32106, Waltham, MA) was used to detect signals. Images were captured using Amersham™ ImageQuant™ 800 (Cytiva, Washington, DC).

### Immunohistochemistry

Mouse liver samples were cut into serial sections (5 µm thick). Deparaffinized sections were subjected to COMT staining using an anti-COMT antibody (Proteintech, Cat# 14754-1-AP, Rosemont, IL). Mouse liver paraffin slides were deparaffinized in xylene twice for 5 min each and soaked in 100%, 95%, or 70% alcohol for 3 min each. After being incubated in 10 mmol·L^−1^ citrate buffer (pH 6.0) for 30 min in a microwave, the slides were incubated in 3% H_2_O_2_ for 10 min at room temperature to block endogenous peroxidase activity. They were then blocked with 5% normal goat serum for 1 h, followed by incubation with anti-COMT antibody (1:100) overnight at room temperature, washing with phosphate-buffered saline, and incubation with SignalStain® Boost IHC Detection Reagent (HRP, Rabbit) (Cell Signaling Technology, Cat# 8114, Danvers, MA) for 30 min at room temperature. The slides were incubated with a DAB liquid substrate system (Sigma‒Aldrich, Cat# D3939, St. Louis, MO) and counterstained with haematoxylin. Subsequently, the slides were dehydrated with alcohol (75%, 95%, 100%, and 100%) for 5 min each and hyalinized with xylene twice. The slides were viewed under a Keyence BZ-X810 microscope (KEYENCE, Osaka, Japan).

### Sirius red staining

Mouse liver samples were cut into serial sections (5 µm thick). Deparaffinized sections were subjected to Picrosirius red staining for collagen using Picro-Sirius Red Stain Kit (ScyTek Laboratories, Cat# PSR-1, West Logan, UT). Five different areas (× 400) were captured in each sample, and positive areas were identified using a Keyence BZ-X810 microscope (KEYENCE, Osaka, Japan).

### In vitro experiments

LX-2 Human Hepatic Stellate Cell Line (Sigma‒Aldrich, Cat# SCC064, St. Louis, MO) was cultured using a high glucose Dulbecco’s Modified Eagle’s Medium (4,500 mg·L^−1^ glucose, Sigma‒Aldrich, Cat# D5671, St. Louis, MO) with 10% Fetal Bovine Serum (Biowest, Cat# S1400, Nuaillé, France), 1X Penicillin–Streptomycin solution (Sigma‒Aldrich, Cat# P4333, St. Louis, MO), and 1X L-Glutamine solution (Sigma‒Aldrich, Cat# TMS-002, St. Louis, MO). LX-2 cells were used for a BrdU assay.

Human Cryo Kupffer cells (Lonza, Cat# HLKC-500 K, Basel, Switzerland) were divided into four groups for investigation: control, 20 nmol·L^−1^ NE, 200 nmol·L^−1^ NE, and 20 nmol·L^−1^ NE + 200 nmol·L^−1^ NMN (n = 8 per group). The cells were cultured using a KuGM™ Kupffer Cell Culture Bullet Kit™ (Lonza, Cat# MKC-500BK, Basel, Switzerland) in a 96-well plate overnight. Subsequently, one group was preincubated with 200 nmol·L^−1^ NMN (DL-normetanephrine hydrochloride, Sigma‒Aldrich, Cat# N7127, St. Louis, MO) for 2 h at 37 °C and 5% CO_2_. After preincubation, NE was added to the medium to a final concentration of 20 nmol·L^−1^ or 200 nmol·L^−1^, and the cells were incubated for 16 h at 37 °C and 5% CO_2_. The culture medium was collected for an IL-6 assay.

### BrdU assay

A BrdU Cell Proliferation ELISA Kit (abcam, Cat# ab126556, Cambridge, UK) was used for the BrdU assay. LX-2 Human Hepatic Stellate Cell Line (Sigma‒Aldrich, Cat# SCC064, St. Louis, MO) was divided into four groups: control group, 20 nmol·L^−1^ NE, 200 nmol·L^−1^ NE, and 1 µmol·L^−1^ NE (n = 12 per group). The cells were plated at 1.2 × 10^4^ cells per well in a 96-well plate and incubated overnight. Subsequently, NE (Tokyo Chemical Industry, Cat# A0906, Tokyo, Japan) was added to the NE-incubated group medium. After 20-h NE incubation, 1X BrdU reagent was added to all groups. The cells were incubated for 4 h with BrdU. After incubation, we performed the assay following the kit protocol. Finally, the absorption was measured using a microplate reader at wavelengths of 450 and 595 nm.

### IL-6 concentration

The concentration of IL-6 in the cell culture supernatant was measured using a Human IL-6 Quantikine ELISA Kit (R&D Systems, Cat# D6050B, Minneapolis, MN). The absorption was measured using a microplate reader at wavelengths of 450 and 570 nm.

The concentration of IL-6 in mouse liver was measured using a Mouse IL-6 ELISA kit (abcam, Cat# ab100713, Cambridge, UK). Protein concentration in liver lysates was adjusted to 10 mg·mL^−1^ with a RIPA Lysis Buffer System (Santa Cruz Biotechnology, Cat# sc-24948A, Dallas, TX). The liver lysates were then diluted sevenfold with 1X Sample Diluent Buffer and used as the assay sample. Subsequent procedures were carried out following the assay protocol.

### Data and statistical analysis

GraphPad Prism software (ver. 10.2, GraphPad Software, San Diego, CA) was used for the statistical analysis. The data are presented as the mean ± SEM. The Student’s *t* test was utilised to compare two groups, and one-way analysis of variance (ANOVA) followed by Tukey’s test was used for comparisons of three or more groups. Statistical significance was defined as a *P* value < 0.05. In all analyses, we excluded outliers using Grubbs’ test, using a significance level of *α* = 0.05.

## Supplementary Information

Supplementary Information.

## Data Availability

The datasets generated during and/or analysed during the current study are available from the corresponding author on reasonable request.

## Abbreviations

αSMA: α-Smooth muscle actin
BP: Blood pressure
BrdU: Bromodeoxyuridine
COMT: Catechol-*O*-methyltransferase
DBA: DBA/2J
E: Epinephrine
EMPA: Empagliflozin
HSC: Hepatic stellate cell
IPGTT: Intraperitoneal glucose tolerance test
KC: Kupffer cell
MASH: Metabolic dysfunction-associated steatohepatitis
MASLD: Metabolic dysfunction-associated steatotic liver disease
Mg: Magnesium
MN: Metanephrine
NAFLD: Nonalcoholic fatty liver disease
NASH: Nonalcoholic steatohepatitis
NE: Norepinephrine
NMN: Normetanephrine
SAH: *S*-Adenosylhomocysteine
SAM: *S*-Adenosylmethionine
SGLT2: Sodium–glucose cotransporter 2
SNP: Single nucleotide polymorphism
T2DM: Type 2 diabetes mellitus
