# Supplementary material for: Empagliflozin reduces liver fibrosis by restoring catechol-O-methyltransferase activity associated with magnesium levels
Source: Sci Rep. 2025 Jul 26;15:27310. doi: 10.1038/s41598-025-12813-x (PMC12297319; doi:10.1038/s41598-025-12813-x)

Supplementary Figure S1

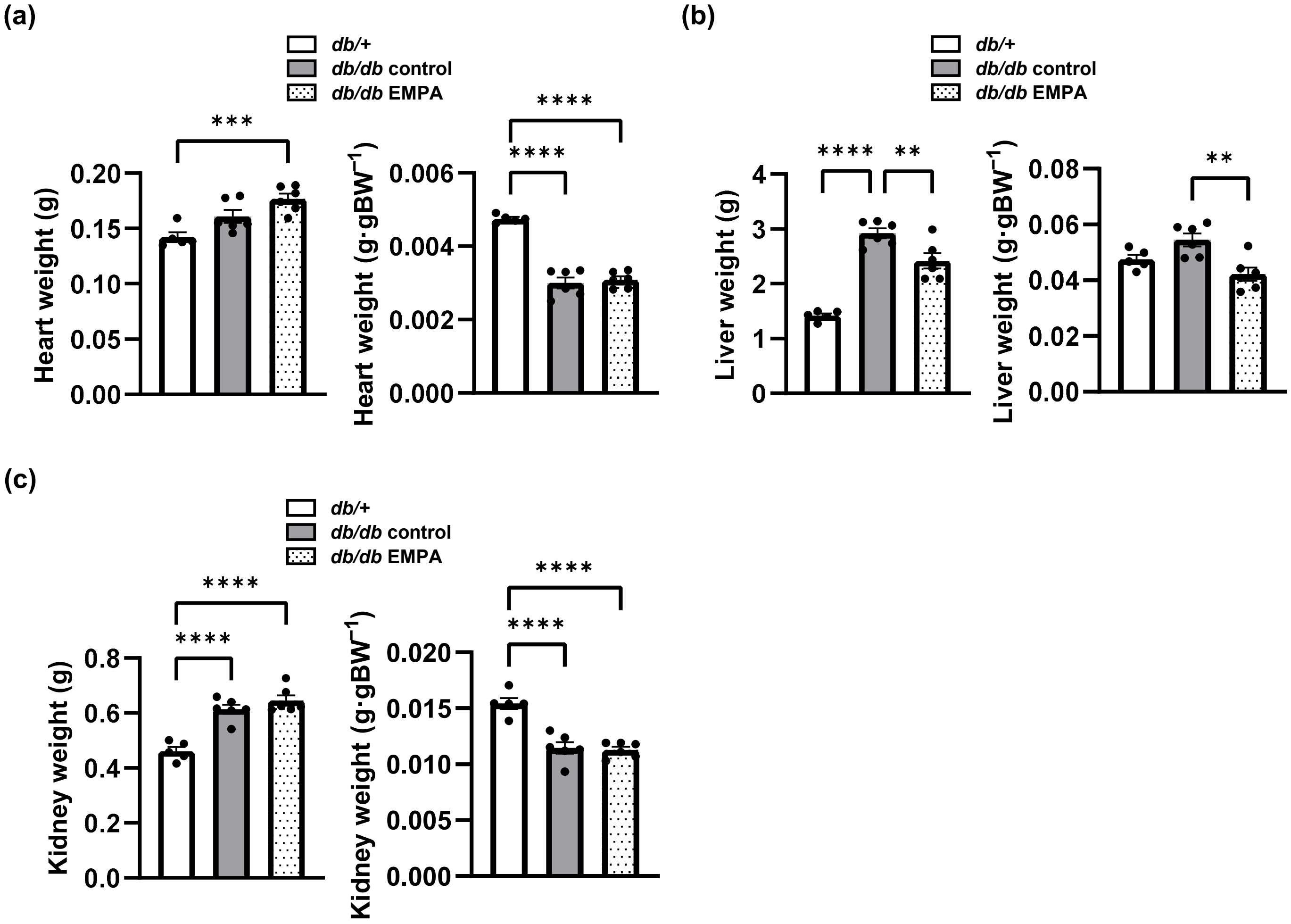

Organ weights of *db/+*, *db/db* control, and *db/db* empagliflozin (EMPA) mice. (a) Heart weight was measured, and heart weight per body weight was calculated in *db/+*, *db/db* control, and *db/db* EMPA mice. (b) Liver weight was measured, and liver weight per body weight was calculated. (c) Kidney weight was measured, and kidney weight per body weight was calculated. *db/+*, n = 5. *db/db* control, n = 6, *db/db* EMPA, n = 6. The data are presented as the mean ± SEM. The data were analysed using one-way ANOVA followed by Tukey's test. **$p < 0.01$, ***$p < 0.001$, ****$p < 0.0001$.

Supplementary Figure S2

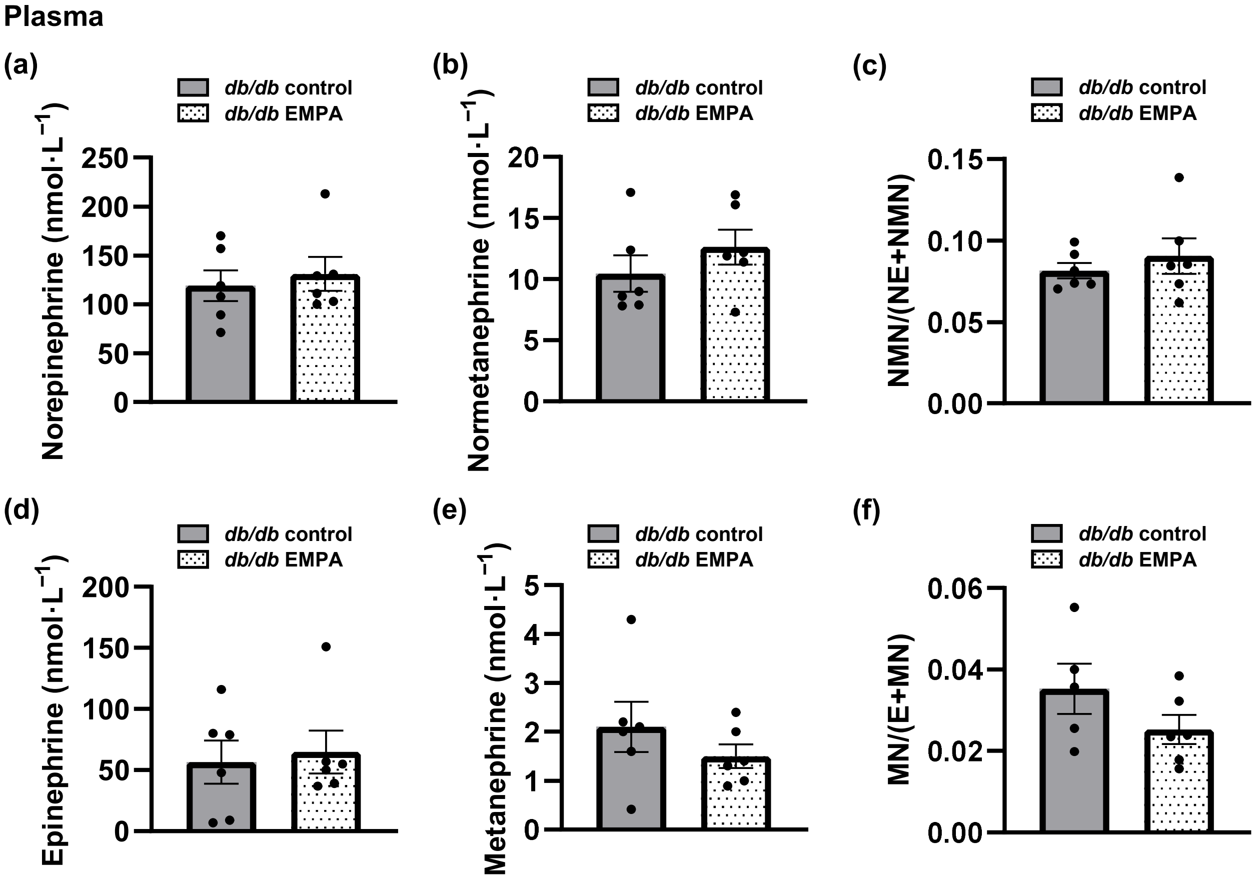

Norepinephrine (NE), epinephrine (E), and metabolite concentrations in plasma. (a) Plasma NE and (b) normetanephrine (NMN) concentrations were measured in *db/db* control and empagliflozin (EMPA) mice. (c) The ratios of NMN to total concentrations of NE and NMN in *db/db* mouse plasma were calculated. (d) Plasma E and (e) metanephrine (MN) concentrations were measured in *db/db* mice. (f) The ratios of MN to total concentrations of E and MN in *db/db* mouse plasma were calculated. *db/db* control, n = 6. *db/db* EMPA, n = 6. The data are presented as the mean ± SEM. The data were analysed using the Student's *t* test.

Supplementary Figure S3

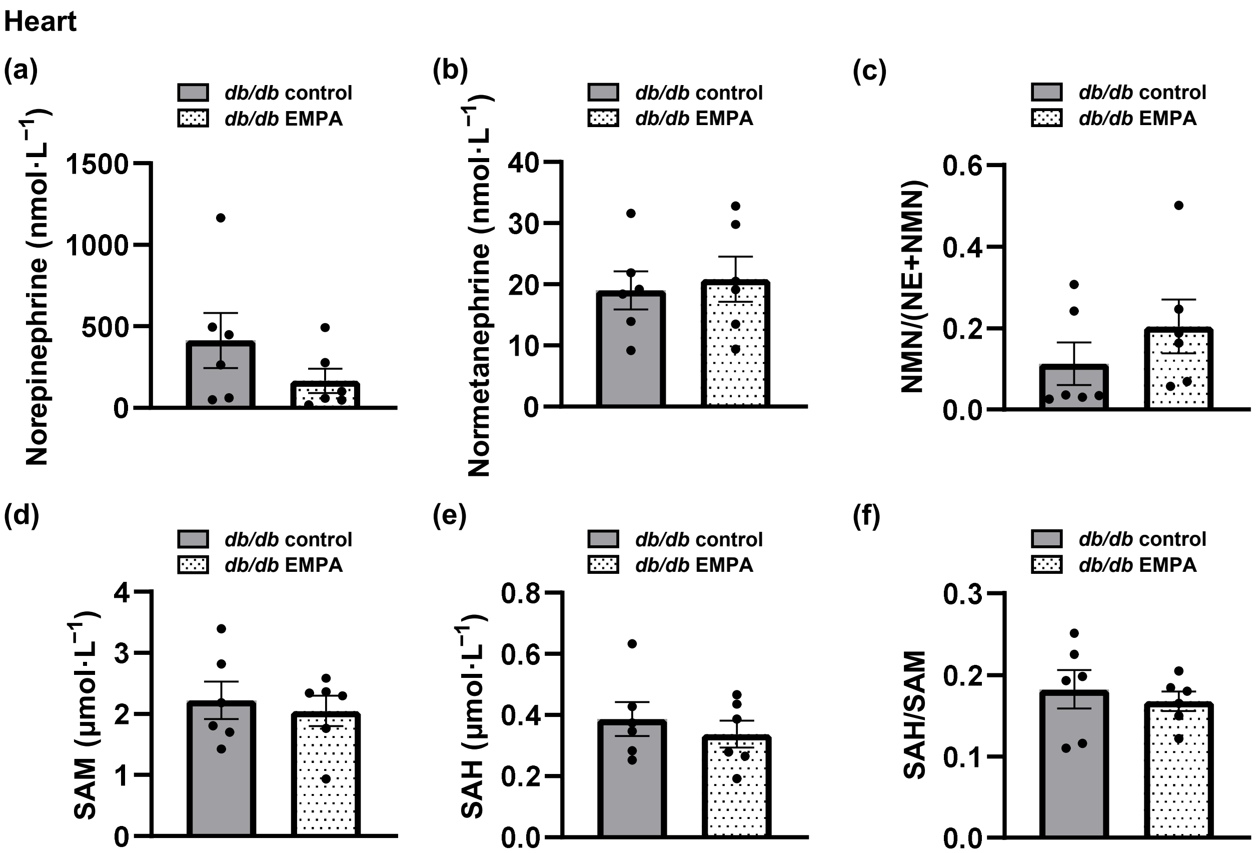

Norepinephrine (NE), normetanephrine (NMN), *S*-adenosylmethionine (SAM), and *S*-adenosylhomocysteine (SAH) concentrations in the heart. (a) Heart NE and (b) NMN concentrations were measured in *db/db* control and empagliflozin (EMPA) mice. (c) The ratios of NMN to total concentrations of NE and NMN in the hearts of *db/db* mice were calculated. (d) Heart SAM and (e) SAH concentrations were measured in *db/db* mice. (f) The ratios of SAH to SAM in the hearts of *db/db* mice were calculated. *db/db* control, n = 6. *db/db* EMPA, n = 6. The data are presented as the mean ± SEM. The data were analysed using the Student's *t* test.

Supplementary Figure S4

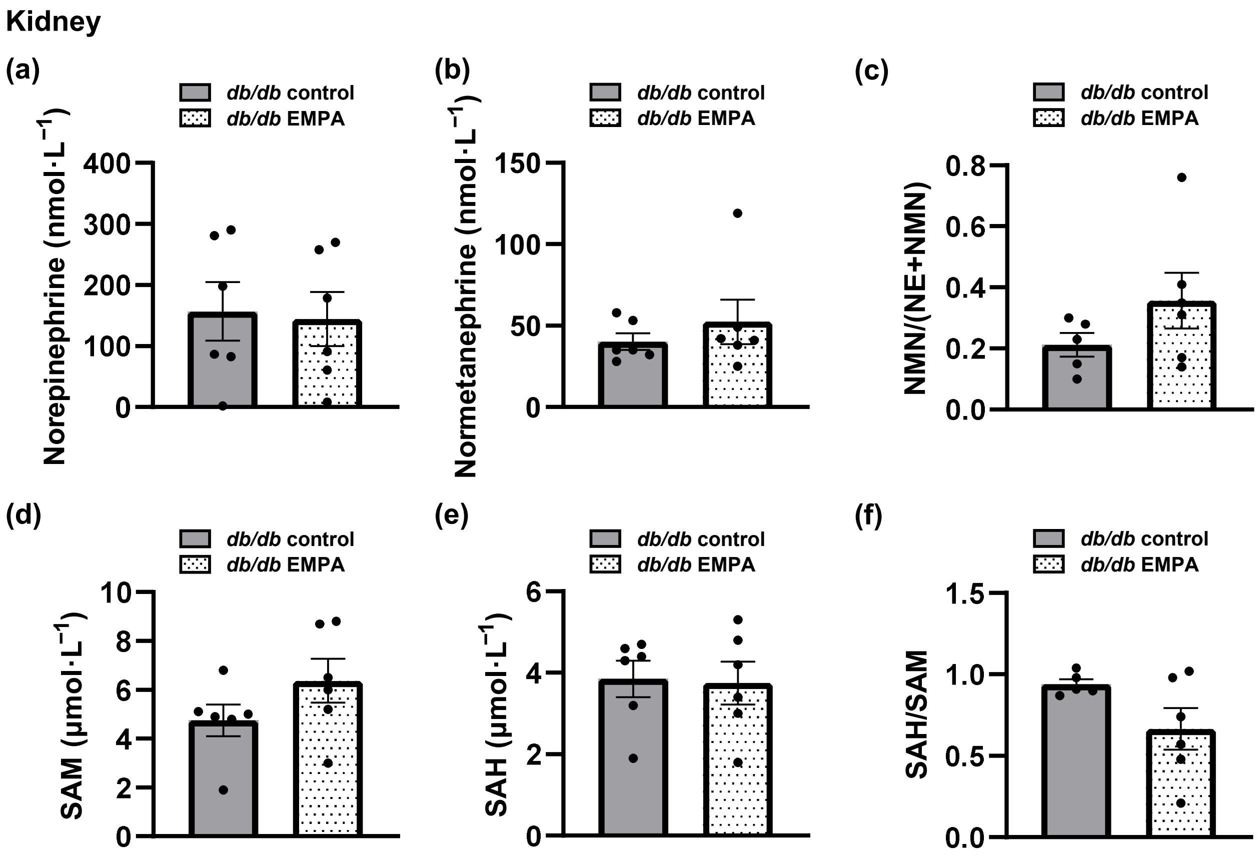

Norepinephrine (NE), normetanephrine (NMN), *S*-adenosylmethionine (SAM), and *S*-adenosylhomocysteine (SAH) concentrations in the kidney. (a) Kidney NE and (b) NMN concentrations were measured in *db/db* control and empagliflozin (EMPA) mice. (c) The ratios of NMN to total concentrations of NE and NMN in the kidneys of *db/db* mice were calculated. (d) Kidney SAM and (e) SAH concentrations were measured in *db/db* mice. (f) The ratios of SAH to SAM in the kidneys of *db/db* mice were calculated. *db/db* control, n = 6. *db/db* EMPA, n = 6. The data are presented as the mean ± SEM. The data were analysed using the Student's *t* test.

Supplementary Figure S5

Original blots

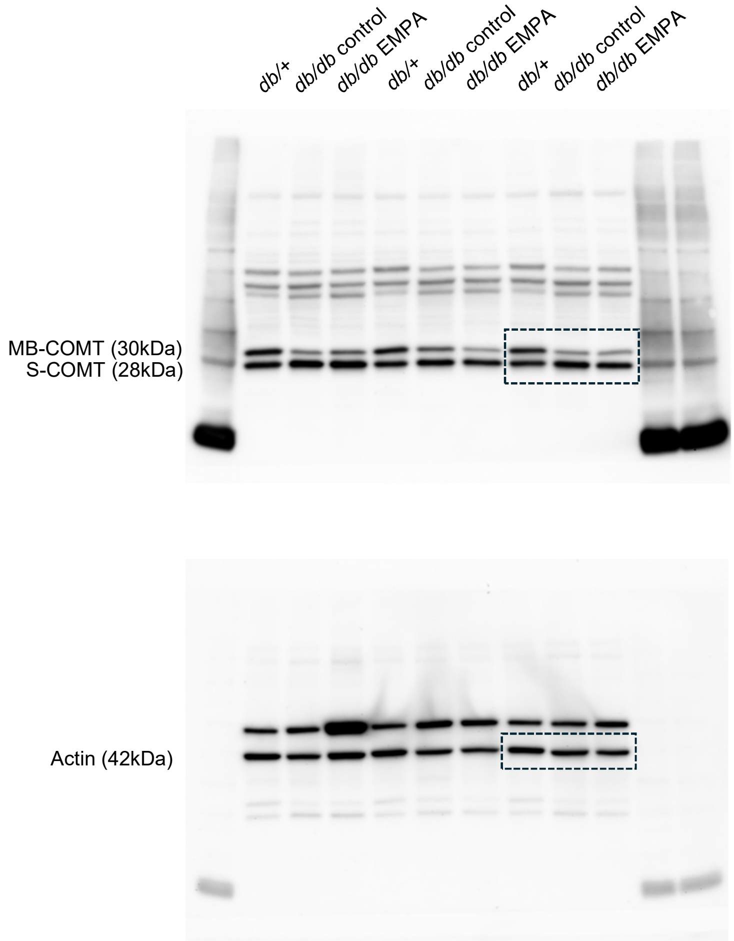

Supplementary Figure S6

Original blots

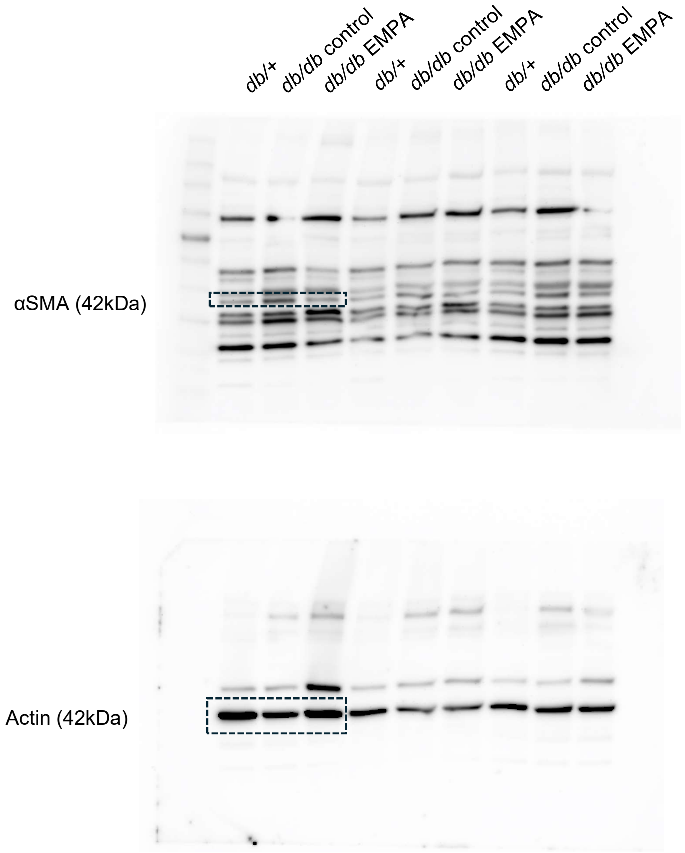

Supplement: Supplementary file 1 — Supplementary Information. [file 41598_2025_12813_MOESM1_ESM.pdf]
